# Computational analysis of local membrane properties

**DOI:** 10.1007/s10822-013-9684-0

**Published:** 2013-10-23

**Authors:** Vytautas Gapsys, Bert L. de Groot, Rodolfo Briones

**Affiliations:** Computational Biomolecular Dynamics Group, Max Planck Institute for Biophysical Chemistry, Am Fassberg 11, 37077 Göttingen, Germany

**Keywords:** Membrane simulations, Molecular dynamics, Area per lipid, Order parameters, Curvature, Bilayer thickness

## Abstract

In the field of biomolecular simulations, dynamics of phospholipid membranes is of special interest. A number of proteins, including channels, transporters, receptors and short peptides are embedded in lipid bilayers and tightly interact with phospholipids. While the experimental measurements report on the spatial and/or temporal average membrane properties, simulation results are not restricted to the average properties. In the current study, we present a collection of methods for an efficient local membrane property calculation, comprising bilayer thickness, area per lipid, deuterium order parameters, Gaussian and mean curvature. The local membrane property calculation allows for a direct mapping of the membrane features, which subsequently can be used for further analysis and visualization of the processes of interest. The main features of the described methods are highlighted in a number of membrane systems, namely: a pure dimyristoyl-phosphatidyl-choline (DMPC) bilayer, a fusion peptide interacting with a membrane, voltage-dependent anion channel protein embedded in a DMPC bilayer, cholesterol enriched bilayer and a coarse grained simulation of a curved palmitoyl-oleoyl-phosphatidyl-choline lipid membrane. The local membrane property analysis proves to provide an intuitive and detailed view on the observables that are otherwise interpreted as averaged bilayer properties.

**Electronic supplementary material:**

The online version of this article (doi:10.1007/s10822-013-9684-0) contains supplementary material, which is available to authorized users.

## Introduction

Studies of biological membrane dynamics play an important role in the field of computational biomolecular simulations. Lipids are known to tightly interact with the proteins embedded in bilayers and affect protein activity [1] acting as cofactors [2, 3] or via non-specific interactions arising due to the bilayer deformation [4–6]. Numerous examples of an inverse effect of proteins perturbing the surrounding lipid shells have been reported [7]. Dynamics of the lipid membrane *per se* is of interest when analysing fundamental biological processes such as vesicle fusion [8] or lipid raft formation [9, 10].

Results of lipid membrane simulations are usually compared with observations from X-ray and neutron scattering or NMR experiments by considering temporal and spatial averages of various observables, e.g. bilayer thickness, area per lipid (APL), deuterium order parameters [11]. The simulation results, however, are not restricted to the average properties, but also allow access to local membrane features the analysis of which may yield valuable insights into the system of interest. The analysis of local membrane properties has contributed to the description of the Aβ peptide’s interactions with the surrounding lipids. Embedded in a bilayer, Aβ was shown to unfold, decrease local membrane thickness and disorder phospholipids around it [12, 13]. Villinger et al. [14] used local membrane thickness calculations to reveal the effect of a single amino acid mutation in the voltage-dependent anion channel (VDAC) on the surrounding lipids. Analysis of local membrane properties has proven to be useful in the description of a fusion inhibitor [15, 16] and in a fusion peptide [17] interactions with a bilayer, as well as in the investigation of cholesterol effects on the lipid structure and dynamics [18, 19].

An algorithm, termed GridMAT-MD [20], was developed to map the lipid atomic positions onto a 2D lattice enabling the efficient calculation of membrane thickness and estimation of the APL including complicated cases where an irregularly shaped protein is inserted in a bilayer. Other approaches for the APL calculation use Voronoi tesselation analysis [21, 22] which can be combined with the Monte-Carlo integration for accurate estimation of the protein occupied area [23]. Several in-house methods for mapping local membrane properties have been reported [18, 24, 25].

The main idea of the current work was to create a comprehensive set of tools for the local membrane property analysis, comprising a collection of methods which allow an efficient and fast calculation of several local membrane properties. These include membrane thickness, APL, acyl chain order parameters and leaflet curvature. The software facilitates membrane property analysis by providing convenient visualization options and a number of choices for the data output. Implementation of the methods in the Gromacs 4.5 [26] framework enables usage of the features native to the *g_tools*, e.g. reading/writing trajectories in all the trajectory formats supported by the Gromacs suite, using index groups for atom selection, distance calculation considering periodic images, analysing user defined fragments of a trajectory, etc. In the course of implementation of the order parameter analysis, we re-derived the equations for the deuterium order parameter (*S*_*CD*_) calculation for unsaturated lipids and improved the solution proposed by Douliez et al. [27] by explicitly considering angles between carbons obtained from simulation. We illustrate the possible applications of this tool-set with a number of examples, namely, a pure dimyristoyl-phosphatidyl-choline (DMPC) bilayer, fusion peptide—part of the GP41 protein—interacting with a DMPC membrane, the VDAC channel embedded in a DMPC membrane, a POPC bilayer enriched with cholesterol and a coarse grained simulation of a curved membrane.

## Methods

### Grid mapping

To map lipid and protein atoms onto a grid we employed a modified version of the GridMAT-MD [20] algorithm. The algorithm is initialized by calculating the center of mass (COM) over a user specified group of atoms, e.g. the whole lipid, head group of a lipid or only the phosphorus atoms, for every lipid of interest. Next the COMs are used to calculate an average coordinate value along the normal to the bilayer axis, which by default is set to be parallel to the *z* axis, but can be changed by the user to *x* or *y* axis if required. Based on the mean COM value the lipids are divided into the top and bottom leaflets of a bilayer. In case a membrane is slightly curved assigning lipids to leaflets based solely on z-coordinates can often fail. We provide an alternative, requiring the user to provide indices of the tail atoms, whereby the lipids are assigned to leaflets based on the relative orientation of the COM of the lipid and the tail atoms. Once the lipids are assigned to their respective leaflets, mapping onto a grid is performed by labeling each grid cell with an index of the lipid closest to it in terms of Euclidean distance.

For the cases where a protein or any other molecule is embedded in the membrane, the procedure to identify protein atoms relevant for the analysis is identical to that described in [20]. Briefly, for every protein atom all the lipids of each leaflet separately are identified within a user defined radius, termed as a ‘precision’ option. A protein atom is allowed to occupy cells on a grid only if there are lipids on one leaflet within the ‘precision’ radius, above and beneath the protein atom. For a graphical illustration of the protein atom inclusion see Fig. 2 in [20]. The identified protein atoms compete for the grid cells based on the distance criteria in the same way as the lipids.

Two methods to determine the grid dimensions have been implemented. A default option uses the box dimensions of every frame if available, otherwise, the dimensions of the first frame are considered. A ‘breathing’ grid option identifies the outermost lipids for every frame and uses their coordinates to define the grid dimensions.

### Membrane thickness

After assigning each lipid or a protein to every grid cell, the membrane thickness is calculated by considering the corresponding pair of cells in the top and bottom leaflets. In the case that both corresponding cells have a lipid assigned, the thickness for the grid element is calculated as a difference between the top and bottom leaflet lipid coordinates along the normal to the bilayer for the lipids assigned to the respective grid cells. For the second case where both corresponding cells have protein atoms assigned, the thickness is defined by a user specified value. For the third case where lipid and protein are assigned to corresponding grid points, the thickness is calculated as a scaled difference between the coordinates of the top and bottom leaflet corresponding cells. The scaling requires a user provided numerical value: *scaled*_*thickness* = *scaling*_*value* × *thickness*. The latter feature allows smoothening the membrane thickness representation in the areas transiently occupied by flexible parts of a protein and enables a more intuitive representation of a protein asymmetrically embedded in the membrane.

### Area per lipid

The local area per lipid is calculated following the algorithm presented in [20]. After mapping the lipids and optionally protein(s) onto a grid, the area occupied by each lipid is estimated by summing the areas of all the cells assigned to the lipid of interest.

The area occupied by protein atoms is also calculated after determining the relevant protein atoms following the procedure described above. The average APL (and standard deviation) of each grid cell and the area occupied by each lipid (as well as the protein area) are reported. In addition, the area of each lipid and/or protein at each time step of a trajectory are provided.

### Acyl chain order parameters

*S*_*CD*_ order parameters are obtained by constructing a molecular frame as described in [28] and defining the order parameter tensor [29]

1 $$S_{ij} = \frac{\langle3 cos \theta_i cos \theta_j - \delta_{ij}\rangle}{2}$$

where θ_*i*_ corresponds to the angle between the bilayer normal and a molecular frame axis (*x*, *y*, *z*). δ_*ij*_ denotes Kronecker’s delta function. For the saturated lipids a relation for the deuterium order parameters is expressed as following [30]

2 $$S_{CD} = \frac{2}{3}S_{xx} + \frac{1}{3}S_{yy}$$

The derivation of Eq. (2) can be found in [27]. The only assumption required to derive the *S*_*CD*_ expression for the saturated lipids is the value of an angle formed by the two deuteriums and a carbon atom to be equal to 109.5°.

**Fig. 1:**
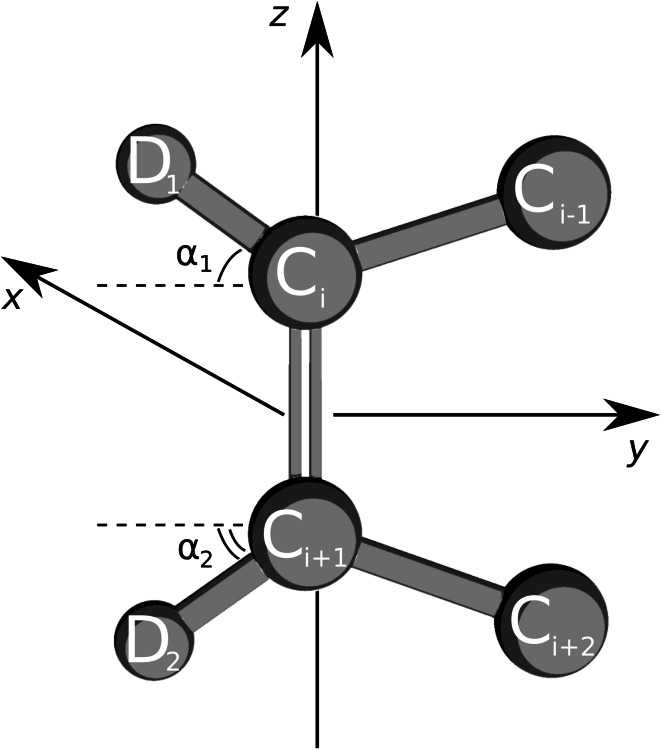
Representation of a *cis* double bond in an unsaturated lipid acyl chain. *x, y* and *z axis* define a molecular frame, *C*_*i*_ denotes carbon atom, *D*_*j*_ depicts deuterium atom

The *S*_*CD*_ relation for deuterium atoms on the carbons involved in double bonds in unsaturated lipids requires different construction of the molecular frame [31] and more assumptions in comparison to the saturated lipids. The *z* axis in the molecular frame for unsaturated lipids (Fig. 1) is defined to be parallel to the double bond. The *x* axis is orthogonal to the plane defined by a deuterium atom and the carbons forming the double bond. The *y* axis is set to be orthogonal to the *z* and *x* axes. Douliez et al. derive a relation (equation (10) in [27]) which requires: a) atoms *C*_*i*−1_, *C*_*i*_, *C*_*i*+1_, *D*_1_ to be in one plane for *S*_*i*_^*CD*^ and atoms *C*_*i*_, *C*_*i*+1_, *C*_*i*+2_, *D*_2_ to be in one plane for *S*_*i*+1_^*CD*^; b) $\angle C_{i-1}C_{i}C_{i+1} = \angle C_{i-1}C_{i}D_1 = 120^{\circ}$ for *S*_*i*_^*CD*^ and $\angle C_{i}C_{i+1}C_{i+2} = \angle C_{i+2}C_{i+1}D_2 = 120^{\circ}$ for *S*_*i*+1_^*CD*^. In fact, the latter constraint can be alleviated by considering the actual angle between the carbon atoms from simulation and placing deuterium such that *C*_*i*_ *D*_1_ and *C*_*i*+1_ *D*_2_ are the angle bisectors. The equations for the order parameters are then written

3 $$\begin{aligned} &S_{C_{i}D_1} = {{\cos}}^2({\alpha_1})S_{yy} + {\sin}^2({\alpha_1})S_{zz} - 2{\cos}({\alpha_1}){\sin}({\alpha_1})S_{yz}\\ &S_{C_{i+1}D_2} = {\cos}^2({\alpha_2})S_{yy} + {\sin}^2({\alpha_2})S_{zz} + 2{\cos}({\alpha_2}){\sin}({\alpha_2})S_{yz} \end{aligned}$$

where $$\alpha_1=1/2(\pi-\angle C_{i-1}C_{i}C_{i+1})$$ and $$\alpha_2=1/2(\pi-\angle C_{i}C_{i+1}C_{i+2}).$$

For the procedure of order parameter mapping onto a 2D grid when a protein is embedded into a membrane, the grid elements that are assigned to a protein are marked by assigning a user specified value. In case where grid cells are transiently assigned to lipids or protein, the averaging considers only those frames where the cell had lipids assigned. Another important notice concerning order parameter mapping is that the lipids compete for the grid cells via the user specified lipid group and not by each acyl chain carbon atom separately.

In addition to the grid mapped *S*_*CD*_ values, order parameters per lipid averaged over time are also reported. To enable faster calculation of the *S*_*CD*_ order parameters for every acyl chain carbon atom, part of the code was parallelized allowing user to specify a number of threads to be used for calculation.

### Curvature

To calculate membrane curvature each grid element is assigned a corresponding lipid or protein coordinate along the normal to the bilayer. First order derivates of the assigned coordinates over the surface *S* = *S*(*x*, *y*) are calculated yielding matrices containing *S*_*x*_ and *S*_*y*_ vectors for every grid cell (*x*, *y*) for both leaflets, where *S*_*x*_ = ∂*S*/∂*x* and *S*_*y*_ = ∂*S*/∂*y*. The first order derivatives are used to calculate the coefficients of the first fundamental form [32, 33]: $E=S_{{x}} \cdot S_{{x}},\, F=S_{{x}} \cdot S_{{y}}$ and $G=S_{{y}} \cdot S_{{y}}.$ To obtain the coefficients of the second fundamental form, firstly, the unit normal to the surface at every grid point is calculated **N** = (*S*_*x*_ × *S*_*y*_)/||*S*_*x*_ × *S*_*y*_||. Afterwards, the second order derivatives, *S*_*xx*_ = ∂^2^ *S*/∂*x* ^2^, *S*_*yy*_ = ∂^2^ *S*/∂*y* ^2^ and *S*_*xy*_ = ∂^2^ *S*/∂*x*∂*y*, over the surface are calculated. This enables estimation of the coefficients of the second fundamental form: $L=S_{{xx}} \cdot {\bf N},\, M=S_{{xy}} \cdot {\bf N}$ and $N=S_{{yy}} \cdot {\bf N.}$ The mean curvature *J* and the Gaussian curvature *K* are calculated

4 $$J = \frac{EN+GL-2FM}{2(EG-F)^2}$$

5 $$K = \frac{LN-M^2}{EG-F^2}$$

Extraction of different spatial frequencies of a curved membrane is possible by performing spectral filtering. For this purpose, the grid-mapped coordinate along the bilayer normal is transformed to Fourier space. A similar approach has been applied for the electron density profile and APL calculation [34]. We employed the fast fourier transform library (fftw [35]) for the 2D coordinate transform. An ideal filter function was used, defined as

6 $$f = \left\{ \begin{array}{ll} 1 & r_{low} \leq r \leq r_{high}\\ 0 & \hbox{otherwise} \end{array} \right.$$

Here *r* defines a fraction of modes to be used. *r*_*low*_ and *r*_*high*_ are user defined values ranging from 0 to 1, controlling filtering properties: *r*_*low*_ = 0 and *r*_*high*_ < 1 corresponds to a low-pass filter, *r*_*low*_ > 0 and *r*_*high*_ = 1 high-pass filter, *r*_*low*_ > 0 and *r*_*high*_ < 1 a band-pass filter (see Figure S2). After the filtering step, an inverse Fourier transform is performed and the recovered coordinate values are used for the curvature calculation. The filtering range can also be provided by using absolute values for the wave vectors, *q*_*low*_ and *q*_*high*_, with the units of nm^−1^. In this case, the filtering range is calculated by considering an absolute number of modes in *x* and *y* directions [34]: $m_q=q\cdot L_x/2\pi$ and $n_q=q\cdot L_y/2\pi$ (Figure S3).

### Output formats

We provide several output formats that can be subsequently used for visualization of the mapped properties. For every selected property, PDB and data matrix files are generated representing the local membrane properties averaged over a trajectory. Atoms in the PDB file correspond to the grid elements of the membrane leaflets averaged over trajectory frames. The average coordinate along the normal to the bilayer is calculated from the atom group selected by the user to represent each lipid molecule. For the order parameter calculation, the coordinate along the normal is calculated for the corresponding acyl chain carbon atoms. Values of a property of interest are stored in the B-factor section of a PDB file. Averaged property values are also stored in the plain text matrix data files separately for the top and bottom leaflets. The matrix entries for the bottom leaflets are mirrored across the *y* axis. The visualization features are summarized in Figure S1. In addition to the averaged values, for the membrane thickness and APL, standard deviations mapped onto a grid are also provided.

A local membrane property of interest, as well as the grid coordinates (as a PDB file) can also be printed out after every frame (as a matrix file) allowing for a kinematic representation. The by-frame output can be smoothed by calculating a running average over a user defined number of frames. We also provide a set of supplementary scripts for manipulation of the data matrix files containing multiple frames and matching them with the corresponding grid coordinate files.

### Example systems

Several systems explored by the means of molecular dynamics simulations were chosen to illustrate the features of the local membrane property analysis software. The selected systems comprised equilibrated systems in the NPT ensemble: a pure membrane patch, a bilayer interacting with a peptide and a protein embedded in a membrane. In addition, the dynamics of a cholesterol enriched phospholipid bilayer was analysed. For the curvature calculation a coarse grained simulation of membrane fusion was used.

#### Pure bilayer

 A 240 ns MD trajectory consisting of 98 DMPC molecules assembled in a lipid bilayer with 3,528 waters was used. The simulation was performed using GROMACS [26] simulation package and the OPLS [36] force-field with the lipid parameters derived by Berger et al. [37]. TIP4P [38] water model was used to solvate the system. A total of 240 ns were simulated with a time step of 4 fs. Neighbor searching was performed every 10 steps. The PME algorithm [39, 40] was used for electrostatic interactions with a cut-off of 1 nm. A cut-off of 1 nm was used for van der Waals interactions. Temperature coupling was done with the v-rescale algorithm [41] at 300 K. Pressure coupling at 1 bar was performed using the Berendsen algorithm [42].

#### Peptide in a membrane

 A system consisting of the first 12 amino acids of the GP41 HIV-1 peptide (fusion peptide) [43] inserted in a patch of 128 DMPC molecules was simulated (Fig. 3). The system contained 6,119 SPC water molecules [44] and sodium and choride ions to a concentration of 150 mM. The GROMOS 43a1 force field [45] was used for the peptide, and the Berger-Gromos port [46] was employed for the lipids. A total of 700 ns were simulated with a time step of 2 fs. The PME algorithm was used for electrostatic interactions with a cut-off of 1 nm. A van der Waals cut-off of 1.4 nm was used. Temperature coupling was done with the v-rescale algorithm at 310 K and the Berendsen algorithm was used for semi-isotropic pressure coupling at 1 bar.

#### Membrane protein

The voltage dependent anion channel VDAC is a β-barrel protein present in mitochondria and important for ATP transport [47]. A system of an mVDAC1 protein (pdb 3EMN) was inserted in a patch of 358 DMPC molecules and simulated for 100 ns as described in [14]. Different variants of an amino acid E73, which is important for the regulation of the protein’s activity [48], were simulated, namely: uncharged E73°, charged E73^−^ and E73V mutant. As the E73 residue points towards the membrane, it makes VDAC an interesting case for the local membrane property analysis.

#### Cholesterol

 Cholesterol induced ordering effects in a lipid membrane were analysed using a 200 ns MD trajectory of the DMPC lipids with cholesterol and TIP4P water from the work by Wennberg et al [49]. The detailed description of the simulation setup and parameters can be found therein.

#### CG-simulation

 An MD simulation of a coarse grained palmitoyl-oleoyl-phosphatidyl-choline (POPC) bilayer was used for the membrane curvature calculations. The whole simulated system constituted of a palmitoyl-oleoyl-phosphatidyl-ethanolamine (POPE) lipid vesicle kept in close proximity to a POPC lipid membrane by a SNARE protein complex (Fig. 6a). The simulated system stayed in a pre-stalk formation phase and the vesicle and bilayer remained unfused. However, the POPC membrane was already curved and had a characteristic ‘dimple’ formed, making it an interesting subject for the local curvature analysis. The more detailed system description and an in-depth analysis of the simulated process can be found in [8].

## Results

### Pure bilayer

**Fig. 2:**
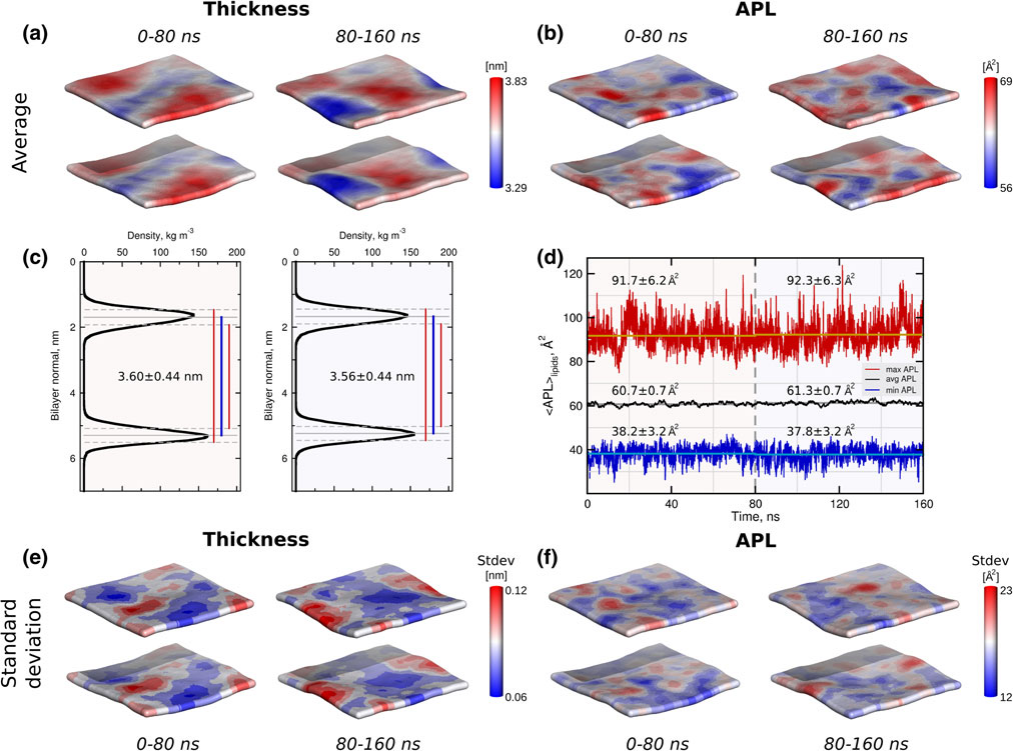
Analysis of the local thickness and APL of a pure DMPC bilayer. A MD simulation trajectory was divided in two 80 ns parts and local properties for both parts were evaluated separately. The area of the box was 5.3 × 5.3 nm^2^ and 100 bins were used along *x* and *y* axes.** a** Local thickness of the bilayer averaged over time. Average thickness values calculated from the grid elements were 3.61 and 3.59 nm for the first and second part, respectively.** b** Local APL of the membrane averaged over time.** c** Phosphorus atom densities averaged over time and over all lipids. Thickness values evaluated from the 1D densities.** d** Change of the APL, averaged over lipids, in time: the* black curve* depicts the mean APL value, the *red* and* blue curves* denote the maximal and minimal APL values, respectively.** e** Standard deviations of the local membrane thickness.** f** Standard deviations of the local membrane APL

To illustrate the general features of the local membrane property analysis we provide thickness and APL calculation of a pure DMPC bilayer simulation (Fig. 2). Phosphorus atoms of the lipid headgroups were considered for both calculations. From a 240 ns trajectory, the first 80 ns were discarded as equilibration and the remaining simulation time was arbitrarily divided in two equal parts in order to assess the convergence and fluctuations of this membrane simulation. Both, the local thickness and APL, were calculated as time averages of the 80 ns trajectory windows (Fig. 2a,b).

The local analysis approach (3.61 and 3.59 nm) corresponds with the global membrane thickness estimation (3.60 and 3.56 nm), which can be calculated from the inter-leaflet peaks of the phosphorus atom densities along the normal to the membrane plane averaging over all the lipids (Fig. 2c). This estimation usually coincides with the inter-leaflet phosphorus peak, as estimated from neutron scattering experiments [50]. While the global analysis shows no changes in the membrane thickness between the two sub-trajectories, local undulations resulting in the thickness changes were revealed by the local thickness calculation.

The APL averaged over all the lipids for a pure phospholipid patch can be extracted from the simulation box area (*xy*) and the total number of lipids. For the MD simulations exploiting a semi-isotropic pressure coupling scheme, changes of the APL over time provide a measure of stability and convergence of the system. The APL time trace can also be extracted using the local APL calculation and averaging over the lipids afterwards, as illustrated by the black curve in Fig. 2d. The APL values calculated from the simulation box dimensions are shown in Fig. S6. An advantage of the local property analysis is the possibility to extract APL values of individual lipids (or protein, if present in the membrane). In Fig. 2d we also depict the minimal and maximal APL values for every time step, which reveals the highly dynamic nature of the lipid density fluctuations. Similarly as shown for the thickness calculations (Fig. 2a), the space averaging can be avoided for the local APL analysis (Fig. 2b). Furthermore, if necessary for a particular study, both, the space and time averaging, can be avoided by calculating the local membrane properties for every frame of a trajectory (see movie in the Supporting Material). In addition to the average properties, estimation of the standard deviations of the local membrane thickness and APL is available (Fig. 2e, f). This feature allows highlighting membrane areas displaying the largest fluctuations.

### Peptide in a membrane

**Fig. 3:**
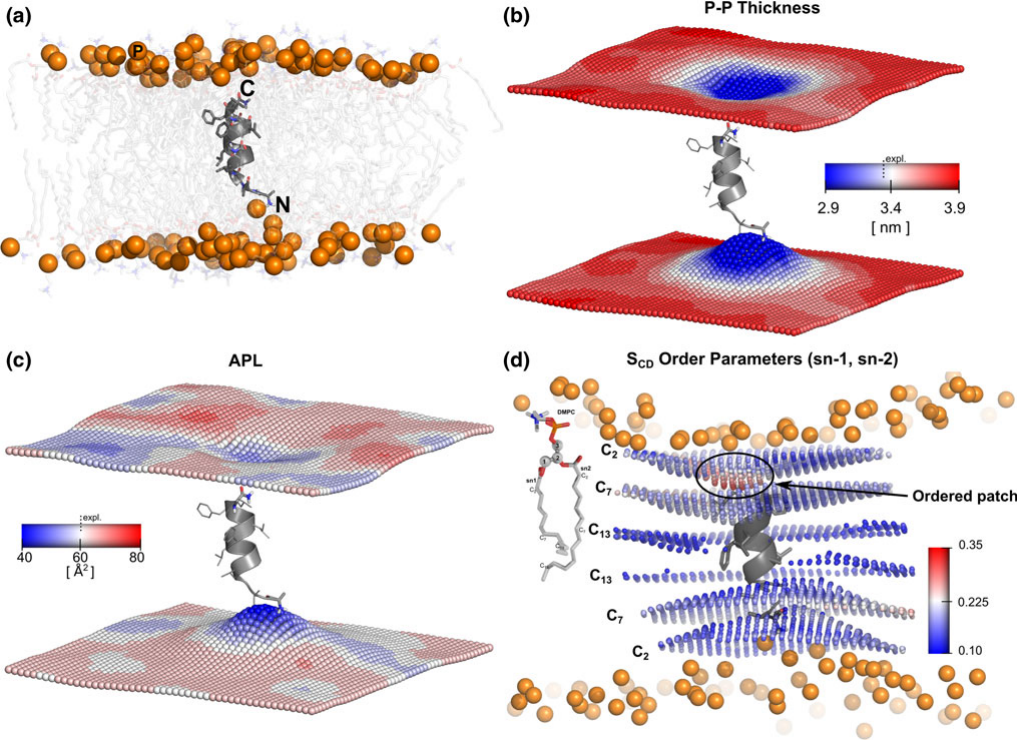
Local properties of a membrane with an inserted fusion peptide. The box area was 6.0 × 6.3 nm^2^ and 50 bins were used along *x* and *y* axes. **a** The first 12 amino acids of a GP41 peptide inserted in a DMPC membrane patch. The peptide is in an α-helical cartoon representation with the amino acid side chains represented as sticks. Lipids are shown as transparent sticks with hydrogens omitted and phosphorus atoms are depicted as spheres.** b** The time averaged local bilayer thickness shows a perturbation of the membrane around the peptide. The experimentally determined thickness of the pure DMPC membrane is shown as a* dotted line* in the* scale bar*.** c** Area per lipid values in each leaflet display an asymmetric distribution of lipids around the peptide. The experimental APL value of pure DMPC is shown as the* dotted line* in the* scale bar*.** d** Local deuterium order parameters of the carbon atoms in positions 2, 7 and 13 of each acyl chain (sn-1 & sn-2) are shown, considering the grid-mapped values within 1 nm of the peptide

The first twelve amino acids of the GP41 protein from HIV-1 are known to function as a minimal fusion peptide. The peptide is able to facilitate lipid mixing and vesicle leakage in vitro [43]. For the current example we used a 700 ns simulation of a single fusion peptide inserted in a DMPC bilayer. The peptide retained an alpha helical structure and oriented almost perpendicular to the membrane plane [51]. The N-terminal end of the peptide remained closer to the lower leaflet as compared to the C-terminal end to the upper leaflet. A snapshot of the peptide orientation in the membrane is shown in Fig. 3a.

In order to rationalize the effects of the peptide on the lipid bilayer we calculated the local thickness, APL and order parameters. Prior to the analysis the system was superpositioned on the peptide backbone in order to eliminate the translational motion of the peptide in the lipid patch.

The local thickness, shown in Fig. 3b, calculated considering the phosphorus atoms, showed the asymmetric perturbation of lipid bilayer caused by the peptide. The positively charged N-terminal part of the peptide distorted the nearby leaflet, as expected for its interaction with the negatively charged phosphate groups. This effect is clearly reflected in the decrease in membrane thickness at the peptide insertion region. The APL depicted in Fig. 3c illustrates the localized effects of a slight increase in lipid density in the vicinity of the peptide. Figure 3d illustrates the *S*_*CD*_ order parameter values and the average positions of the carbons C2, C7 and C13 of the both acyl-chains, sn-1 and sn-2. It can be observed that the carbon atoms closer to the phosphate groups (C2 > C7 > C13) have higher deuterium order parameter values, as expected from the macroscopic (space averaged) *S*_*CD*_ values. Furthermore, our analysis reveals that ordered lipids show a non-homogeneous distribution around the peptide. As emphasized in Fig. 3d, the peptide appears to induce local order in a small patch in one of the leaflets around the C2 carbons.

### Protein in a membrane

**Fig. 4:**
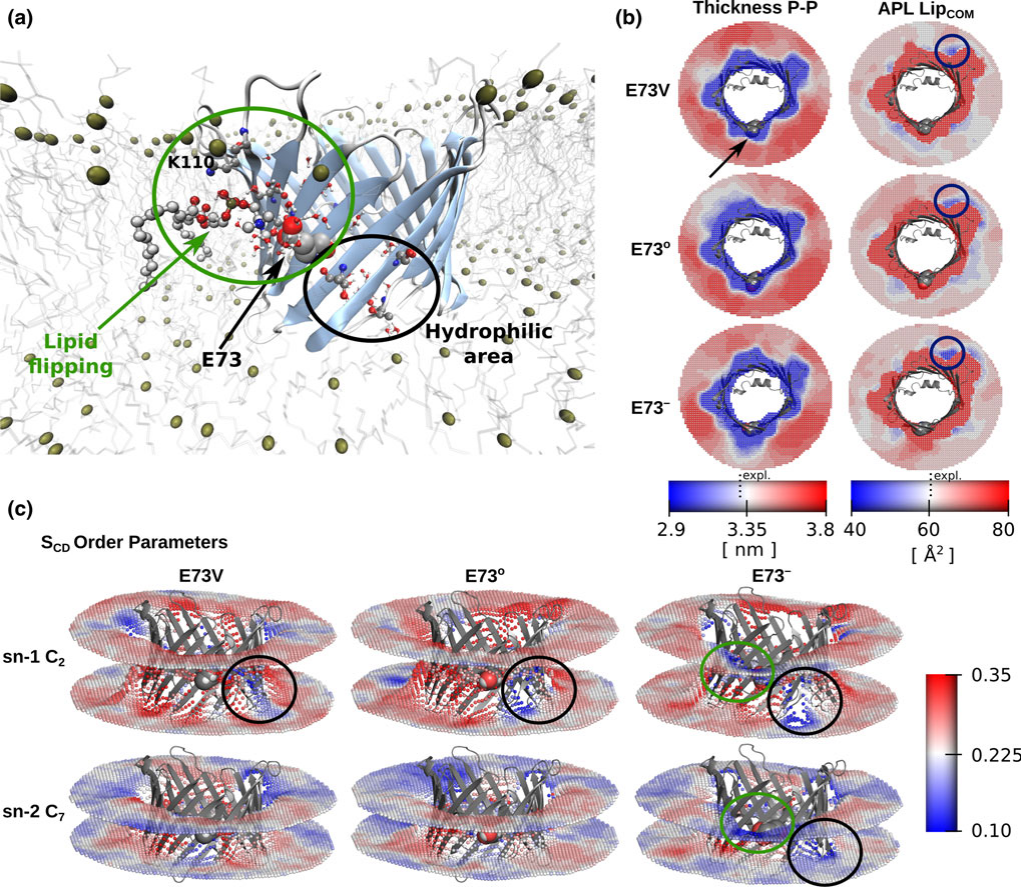
Local membrane properties of a bilayer with an embedded membrane protein VDAC.** a** The voltage dependent anion channel (VDAC) is shown in a cartoon β-barrel representation. Residue E73 and the water molecules nearby are represented as spheres. Serine and threonine residues constituting hydrophilic area close to E73 are shown in ball and stick representation. In the snapshot, a DMPC lipid is shown flipping close to the E73 and K110 residues. The box area was 11.5 × 11.5 nm^2^ and 100 bins were used along *x* and *y *axes.** b** Top view perspectives of the circular representation of the local thickness, calculated considering phosphorus atoms, and area per lipid, calculated considering the COMs of the lipids. The properties were estimated for the simulations of the E73V, uncharged E73° and charged E73^−^ variants of VDAC. The* arrow* indicates the position of E73X residue (sphere representation).** c** Circular representation of the deuterium order parameters for selected carbons around VDAC

The glutamate residue in position 73 in mammalian VDAC plays an important role in the regulation of VDAC’s function [48]. Simulations together with NMR experiments [14] suggested that the structural fluctuations of the protein are compatible with E73 in a charged state. Also, the local membrane thickness around this position was affected by the type of amino acid present there [14]. Previously reported results of the MD simulations revealed phospholipid flipping around E73 and K110 and transient water entry on the opposite bilayer around a hydrophilic patch of Ser and Thr residues, as highlighted in Fig. 4a. Here we extended the local membrane property analysis to the APL and *S*_*CD*_ using the simulations carried out for the charged E73^−^, uncharged E73° and E73V variants of VDAC-1.

In Fig. 4b a comparison of the APL and membrane thickness are presented. Phosphorus atoms were selected to represent the lipids for the thickness calculations and the backbone atoms to represent the protein. Whereas for the APL estimation the COM of a whole lipid was considered. To avoid protein rotation, trajectories were superimposed on the protein backbone atoms. The thickness representation showed equivalent results to those described by Villinger et al. [14], where a charged E73^−^ residue distorted a larger membrane area in comparison with the other conditions, neutral E73° and E73V. The APL estimates were qualitatively similar for all the E73 variants. The low APL area, corresponding to the more localized lipid region, close to the Trp210 residue (marked in Fig. 4b) matches also the position of a co-crystallized DMPC molecule in the VDAC structure at 2.3 Å [52].

Figure 4c depicts the local deuterium order parameters for the C2 and C7 carbon atoms of the sn-1 and sn-2 acyl chains. Here we observed that the *S*_*CD*_ values distribute inhomogeneously around the protein and between the bilayer leaflets. It was possible to identify areas where the lipid ordering was locally disrupted. All the E73 variants showed low C2 carbon *S*_*CD*_ values around the hydrophilic patch, whereas C7 carbons exhibited considerable distortion for the charged E73^−^ residue. The charged E73^−^ residue also perturbed the lipids at the lipid flipping area in both leaflets.

### Cholesterol

**Fig. 5:**
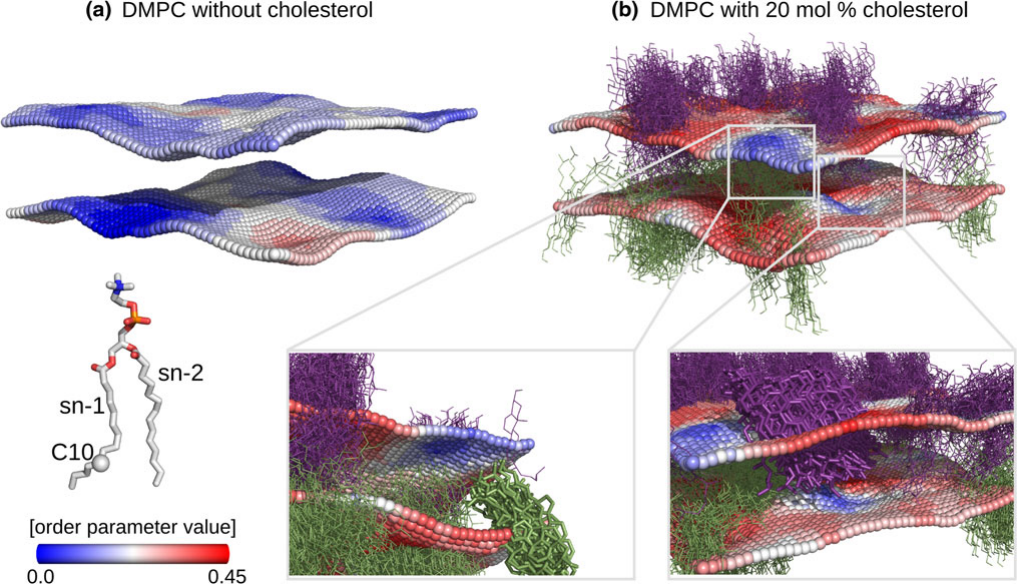
*S*_*CD*_ order parameter analysis of a cholesterol enriched DMPC bilayer. Order parameter values for the C10 carbon of the acyl chain sn-1 are shown. The box area was 5.6 × 5.6 nm^2^ and 50 bins were used along *x* and *y *axes.** a** Local deuterium order parameters of a pure DMPC membrane averaged over a 100 ns MD trajectory.** b** Local *S*_*CD*_ order parameters for a 20 mol% DMPC bilayer averaged over a 10 ns MD trajectory excerpt. The cholesterol molecules represented as* lines* are overlayed for all the frames. The enlarged view figures emphasize the areas where cholesterol (visualized as sticks) decreases ordering of the lipid chains on the opposite leaflet

The local order parameter analysis was applied to a DMPC lipid membrane containing 20 mol% of cholesterol, which corresponds to a 100 phospholipid and 24 cholesterol molecules in a simulation box. For the analysis, the last 10 ns of a longer 200 ns trajectory were used and the mapped order parameter values were averaged over time (Fig. 5b). In Fig. 5 the order parameters of the carbon atom (C10) of the acyl chain sn-1 are depicted. Cholesterol molecules for all the frames of 10 ns trajectory are shown. Additionally, an analysis of a DMPC lipid membrane without cholesterol was performed, where the mapped order parameter values were averaged over a 100 ns MD trajectory (Fig. 5a).

The cholesterol induced phospholipid ordering effect, which has been observed and well described in both experimental [53–56] and computational [57–61] studies, is clearly visible in the color-coded bilayer representations. Mapping of the local order parameter values, however, allows to gain a deeper insight into the effects the cholesterol has on the acyl chain ordering. It appears that the cholesterol containing membrane has *S*_*CD*_ values of ∼0.3 and larger even in the regions where a cholesterol molecule does not diffuse during the period of observation. A peculiar feature that is emphasized in the enlarged excerpts in Fig. 5b, hints that cholesterol may have an effect on the order of the carbon atoms of an opposite leaflet. The lowest order parameter values were observed in the area not occupied by the cholesterol molecules, but containing cholesterol in the opposite leaflet. The stiff cholesterol molecule, while inducing order in the phospholipids on the same monolayer, appears to distort the lower parts of the acyl chains of the lipids on the other leaflet. This observation comes in accord with the previously reported simulation results of cholesterol being able to intercalate between the membrane leaflets [62] and the experimental findings reporting on the stronger cholesterol ordering effect on the lipids with longer acyl chains [63].

### Coarse grained membrane

**Fig. 6:**
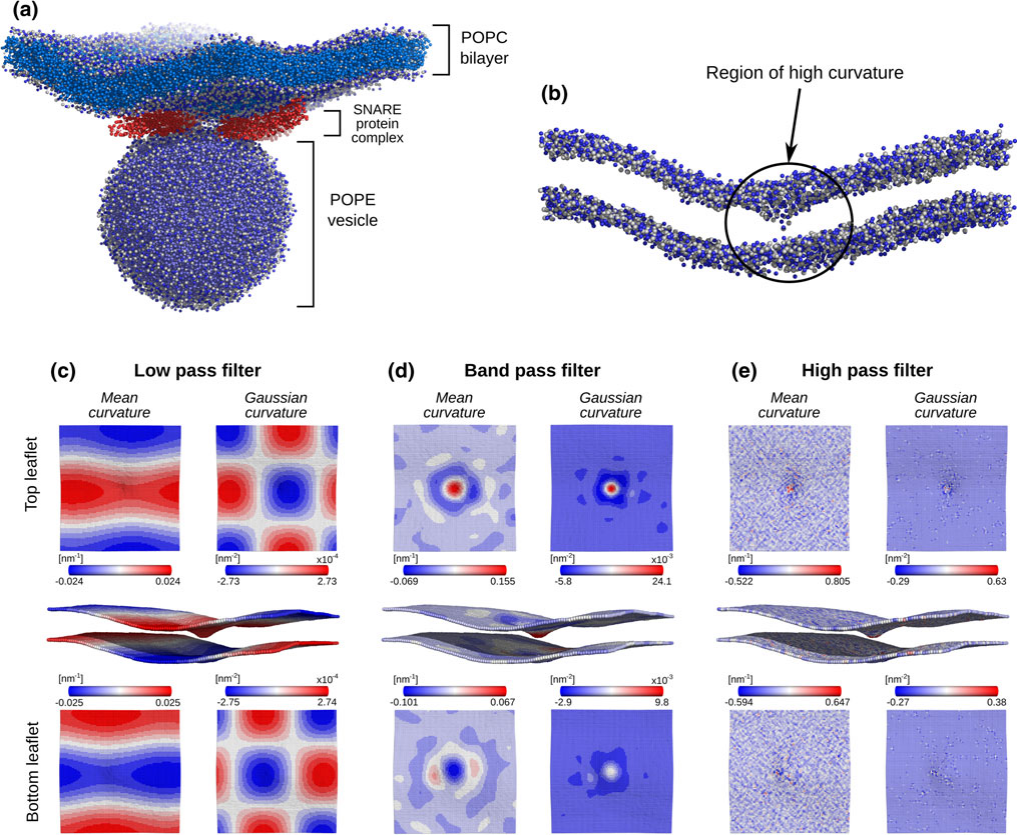
Curvature of a coarse grained POPC bilayer simulation.** a** The whole simulated system containing a SNARE protein complex holding a POPE lipid vesicle and POPC bilayer in a pre-stalk formation phase. The box area was 30 × 30 nm^2^ and 100 bins were used along *x* and *y *axes.** b**. A POPC bilayer with only the beads representing the amine (*blue*) and phosphate (*gray*) groups is shown. The characteristic high curvature ‘dimple’ region is highlighted.** c** Gaussian and mean curvatures after applying a low pass filter.** d** Gaussian and mean curvatures after applying a band pass filter.** e** Gaussian and mean curvatures after applying a high pass filter

A POPE lipid vesicle and POPC lipid bilayer in the pre-stalk formation stage served as a system for the local curvature analysis. We used a 44 ns fragment of a coarse grained MD trajectory considering beads representing phosphate groups for the calculations. As can be seen in a snapshot of the system (Fig. 6a), the bilayer is slightly bent, hence having an overall low curvature. Additionally, a small patch in the middle of the membrane forms a ‘dimple’ of high curvature (Fig. 6b). Using the local membrane property analysis we were able to capture different curvature modes, as illustrated in Fig. 6c–e. The low frequency modes (Fig 6c) were obtained by applying a low pass filter discarding all frequencies higher than the ones defined by the radius *q*_*low*_ = 0.25 nm^−1^ in the frequency domain. While the mean curvature illustrates the overall bending of the bilayer (also visible in the snapshot), the gaussian curvature captures more subtle low frequency undulations of the membrane resulting from the changes of the principal curvature signs. The band pass filtered (*q*_*low*_ = 0.5 nm^−1^, *q*_*high*_ = 1.0 nm^−1^) trajectory (Fig. 6d) captures the strongly curved region of the bilayer, whereas processing with the high pass filter (*q*_*low*_ = 1.0 nm^−1^) leaves only high frequency noise (Fig. 6e).

## Discussion

A number of recent applications of the local property analysis for lipid membranes proved to yield new insight into biological processes [12–14, 18, 25]. In the current work we describe a unified framework for the local membrane property calculation. The methods presented here allow for an efficient mapping of the local membrane thickness, APL, deuterium order parameters, Gaussian and mean curvatures. Using a number of different membrane systems, we covered a diverse set of applications, where the local properties could provide a better understanding of the lipid dynamics and interactions.

The local property analysis exploits the high level of detail obtainable via MD simulations. While experimental measurements are often limited to space and/or time averages, simulation trajectories do not suffer from such restrictions. As illustrated by the pure DMPC lipid patch analysis, the local bilayer thickness and APL calculations complement averaged property estimates and clearly reveal the inherently dynamic nature of lipid bilayers. A powerful feature of the APL extraction for the individual lipids at every time step illustrates that, although the average APL fluctuates only mildly, the minimal and maximal APLs can significantly deviate from the mean value. The calculation of the local membrane thickness and APL can provide an additional measure for the convergence of a simulation. A usually applied convergence criterion is an APL value leveled off over time. However, following the ergodicity principle, for a well equilibrated system, time averages are expected to converge to the spatial averages. Hence, the usual convergence test can be complemented by monitoring the local membrane properties.

The local property analysis allows choosing any representative atom (or group of atoms) for the analysis. Currently the user should specify which atoms are meaningful (headgroup, glycerol, acyl chain atoms, whole lipid, etc) for the specific property calculation. As shown in the example of Fig. S5 it is possible to get meaningful results for the APL of a mixed bilayer containing a mixture of small and large lipids. The simulation of Cholesterol-DPPC-POPC (1:2:2) shows that atoms forming slightly different layers in the membrane can be chosen. This area estimation can be done without any a priori information (cross section area or molar volumes in the case of cholesterol [49, 64]) and it can be useful for studying multicomponent lipid mixtures.

For the membrane system interacting with a short peptide, local thickness and APL calculations provided intuitive means of measure and visualization of the basic effects the peptide exerted on the bilayer. A more intriguing feature was revealed by the deuterium order parameter analysis: a localized leaflet area near the C-terminal end of the peptide showed an increase in the lipid ordering. This preliminary observation, though requiring a further in-depth analysis, hints at a tight lipid-peptide interaction, which could be verified by a mutation analysis and additional MD simulations.

**Fig. 7:**
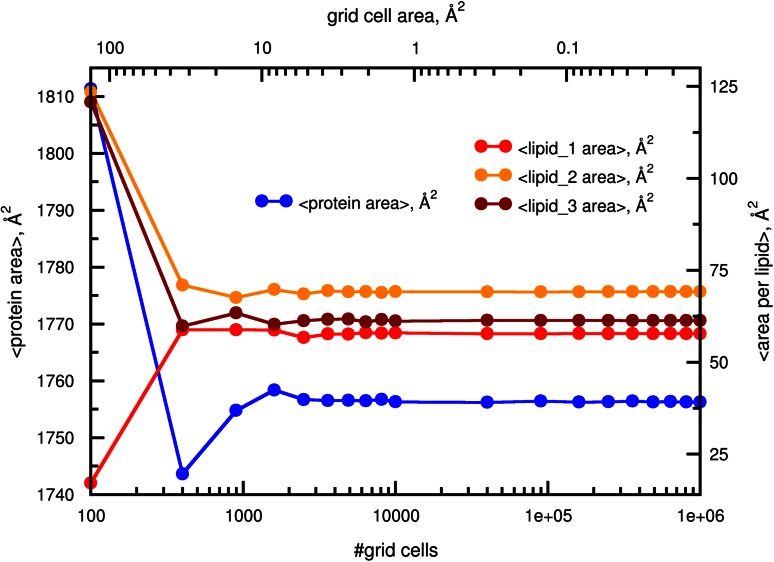
Dependence of the calculated area per lipid/protein on the user defined grid cell number. A molecular dynamics trajectory of a protein VDAC embedded in a DMPC bilayer was analysed by calculating local area per lipid/protein using different number of grid cells. Both, the membrane area occupied by the protein and the local APL of three randomly selected lipids converge for the grid cells with area smaller than 10 Å^2^

The results of any grid based approach depend on the fineness of the utilized lattice. While finer grids yield more accurate mapping, the computational cost increases with the number of grid cells that need to be considered. To provide an estimate of a suitable number of grid cells to use for the local membrane property calculation, we calculated an average local APL for the DMPC bilayer with an embedded VDAC protein from a 100 frame trajectory (Fig. 7). The APL for three randomly selected lipids and the area occupied by the protein were monitored for the different grid sizes. The estimated areas converge for the grid cell sizes of 1−10 Å^2^, which in turn can be considered a reasonable choice for the local membrane property analysis. The grid size effects are also visualized in the Supporting Information Figure S4: for a single frame APL analysis, the decrease in the area per grid cell enforces formation of the Voronoi tesselations (this effect has also been reported in [20]). It comes as a result of the grid mapping procedure, where every lipid competes for grid cells based on a Euclidean distance criterium. While using grid cells of <1 Å^2^ does not have a significant effect on the local membrane property estimates (Fig. 7), finer grids may prove useful for generating high resolution membrane images (Fig. S4).

The analysis of the MD simulations of the membrane protein VDAC embedded in a bilayer represents another important area of application for the local membrane property estimation. As already demonstrated by Villinger et al. [14] and successfully reproduced in the current study, introduction of a charge on the E73 residue of VDAC results in a reduced bilayer thickness in the vicinity of E73. Calculation of the local *S*_*CD*_ order parameters provides more direct evidence of the disordering effect the negatively charged glutamate 73 residue has on the surrounding lipids. It is important to mention the influence that superpositioning has on the local property estimates. In case, a trajectory is superimposed on the protein atoms only, at every time step the bilayer will be translated and rotated following the translation/rotation of the protein. Local membrane properties calculated for a trajectory pre-processed in such a way will suffer from the inaccuracies at the edges of the simulation box. An elegant way to circumvent this problem is concentrating on the areas unaffected by the superpositioning artefacts, e.g. using circular representations discarding the smeared out regions of the membrane, as illustrated in Fig. 4b, c.

Analysis of the *S*_*CD*_ order parameters of a cholesterol enriched DMPC bilayer proved that the local property analysis can be a powerful method for studying cholesterol induced effects. As discussed in the review by Róg et al. [65], cholesterol has a stronger ordering effect for the longer acyl chain lipids in comparison to the shorter lipids. The explanation for this observation comes from the fact that cholesterol is able to intercalate between the leaflets for the shorter phospholipid bilayers. The local property analysis is able to capture the details of this process. As we were able to precisely pinpoint the locations of the disordered regions, the cholesterol appeared to distort the acyl chain ends of the lipids in an opposite leaflet.

By the example of a coarse grained POPC membrane, we illustrated the capabilities of our methods to capture membrane undulations of various frequencies in terms of local curvature. The current implementation of the methods deals only with relatively flat membranes, which poses an obvious limitation for the analysis of such biologically and physically interesting processes as vesicle fusion without a prior pre-processing of the trajectories. An interesting graph based *LeafletFinder* algorithm described by Michaud-Agrawal et al. [66] offers an attractive alternative that could allow extending the local membrane property mapping for highly curved membranes.

The local membrane property analysis is not limited to the methods presented in the current study. The software can be extended for other lipid properties of interest. It is also important to note, that, although, in the current study we mostly emphasized the calculation of spatially non-averaged properties, the presented methods can be used for the average property calculation. Thus, the software can also be used for the calculation of the average APL or *S*_*CD*_ order parameter values.

## Conclusions

The software for local membrane property analysis allows exploiting the high level of detail available from the MD simulations. It is applicable to a wide range of membrane systems for the calculation of the local thickness, APL, deuterium order parameters and bilayer leaflet curvature. The analysis enables detection of spots of interest around proteins or peptides embedded in membranes.

The software and source code is developed as a Gromacs analysis tool and is freely available from http://www.mpibpc.mpg.de/groups/de_groot/software.html.

## Electronic supplementary material

Below is the link to the electronic supplementary material.

PDF (3798 KB)

### Electronic supplementary material

MPG (47750 KB)
