# Supplementary material for: Computational analysis of local membrane properties
Source: J Comput Aided Mol Des. 2013 Oct 23;27(10):845–58. doi: 10.1007/s10822-013-9684-0 (PMC3882000; doi:10.1007/s10822-013-9684-0)
Supplement: Supplementary file 1 — PDF (3798 KB) [file 10822_2013_9684_MOESM1_ESM.pdf]

Supporting Information: Computational Analysis of Local Membrane Properties

Vytautas Gapsys · Bert L. de Groot · Rodolfo Briones

Vytautas Gapsys
Computational biomolecular dynamics group,
Max Planck Institute for Biophysical Chemistry,
Am Fassberg 11,
Göttingen 37077, Germany
Tel.: ++49-551-2012310
Fax: ++49-551-2012302
E-mail: vgapsys@gwdg.de

Bert L. de Groot
Computational biomolecular dynamics group,
Max Planck Institute for Biophysical Chemistry,
Am Fassberg 11,
Göttingen 37077, Germany
Tel.: ++49 551-201-2308
Fax: ++49-551-2012302
E-mail: bgroot@gwdg.de

Rodolfo Briones
Computational biomolecular dynamics group,
Max Planck Institute for Biophysical Chemistry,
Am Fassberg 11,
Göttingen 37077, Germany
Tel.: ++49-551-2012309
Fax: ++49-551-2012302
E-mail: rbrione@gwdg.de

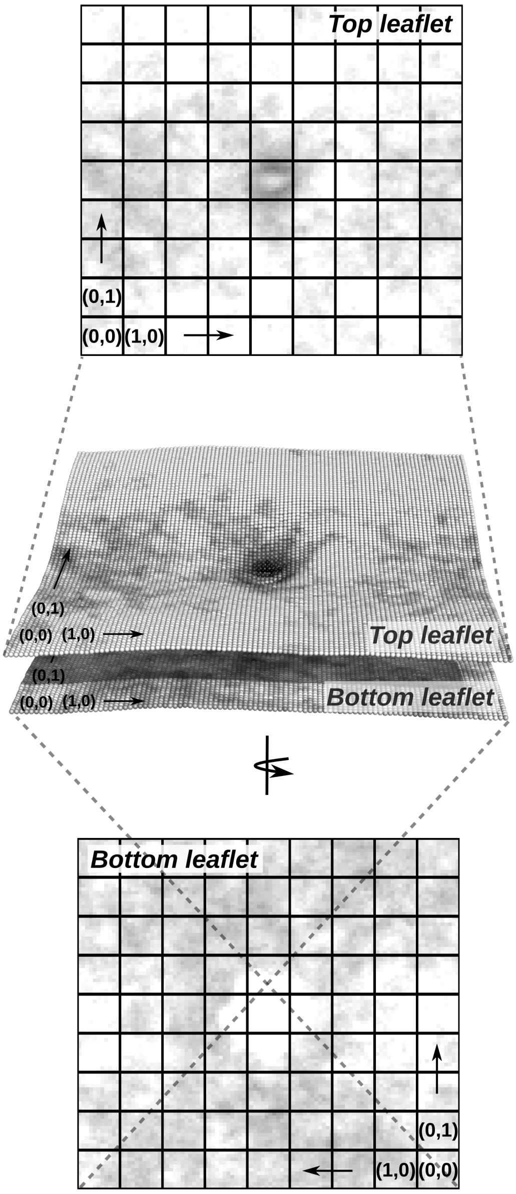

Fig. 1 Summary of the visualization formats for the computed local membrane properties. A time average property is stored in the B-factor section of a pdb file. The atom coordinates in the pdb file correspond to the grid cells and match the representative lipid coordinate along the bilayer normal. The properties are also provided as matrices in a plain text format, for the top and bottom leaflets separately. The bottom leaflet representation in the plain text data matrix file is rotated by 180°, as illustrated in the figure, allowing the visualization of the leaflet as if the bilayer was flipped over.

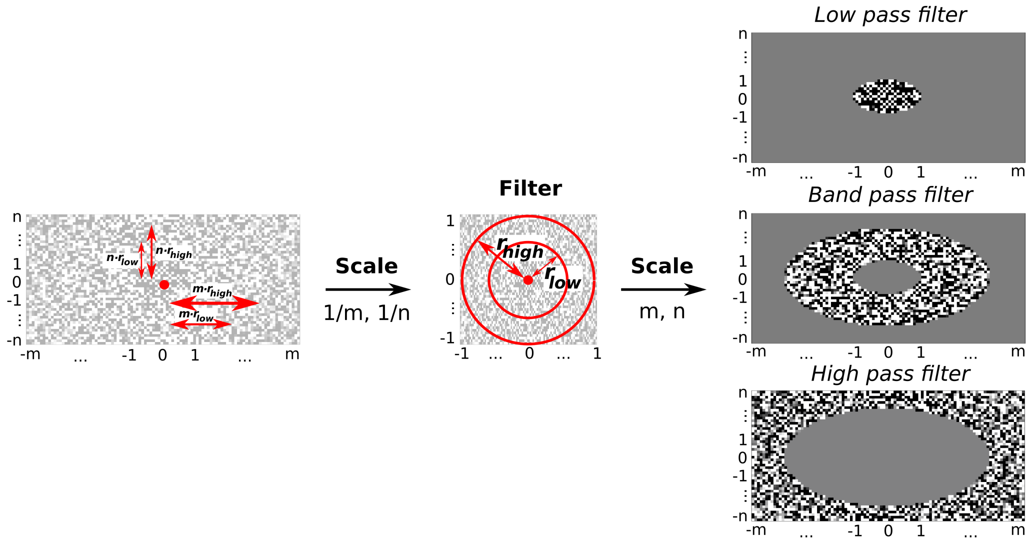

Fig. 2 Schematic illustration of the filtering procedure when a relative number of modes is provided. A Fourier transformed single leaflet along the normal to the bilayer is depicted in the image on the left. The coordinates denote modes in the reciprocal space. A rectangular 100x50 grid was used for the property mapping. In the first step the grid is scaled to range from -1 to 1 in both dimensions. The filtering is applied using an ideal filter controlled by the two radii, r_{high} and r_{low}, as described in the main text. Once scaling is performed the radii in x and y directions are equal, hence the filtered region is represented by a circle. After the filtering is performed, the grid is re-scaled back to the original dimensions. When relative number of modes is used for filtering, the filtered region depends on the r_{high} and r_{low} values as well as on the grid dimensions.

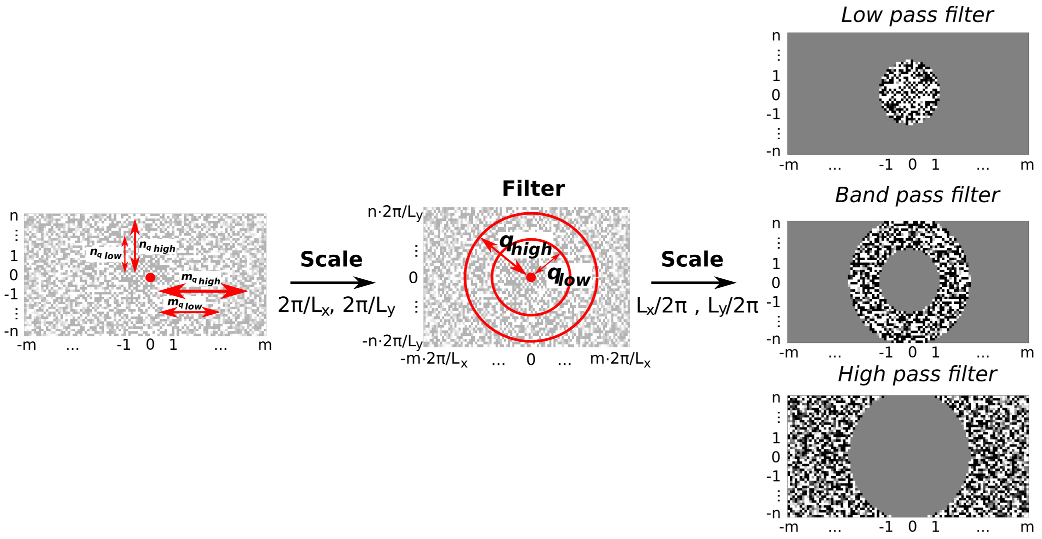

Fig. 3 Schematic illustration of the filtering procedure when absolute cut-off values are provided. A Fourier transformed single leaflet along the normal to the bilayer is depicted in the image on the left. The coordinates denote modes in the reciprocal space. A rectangular 100x50 grid was used for the property mapping. In the first step the grid is scaled to range from $-m \cdot 2\pi/L_x$ to $m \cdot 2\pi/L_x$ and from $-n \cdot 2\pi/L_y$ to $n \cdot 2\pi/L_y$ in x and y directions, respectively. The filtering is applied using an ideal filter controlled by the two radii, q_{high} and q_{low} (units of nm^{-1}), as described in the main text. The user defined q values are equal for x and y directions. After the filtering is performed, the grid is re-scaled back to the original dimensions.

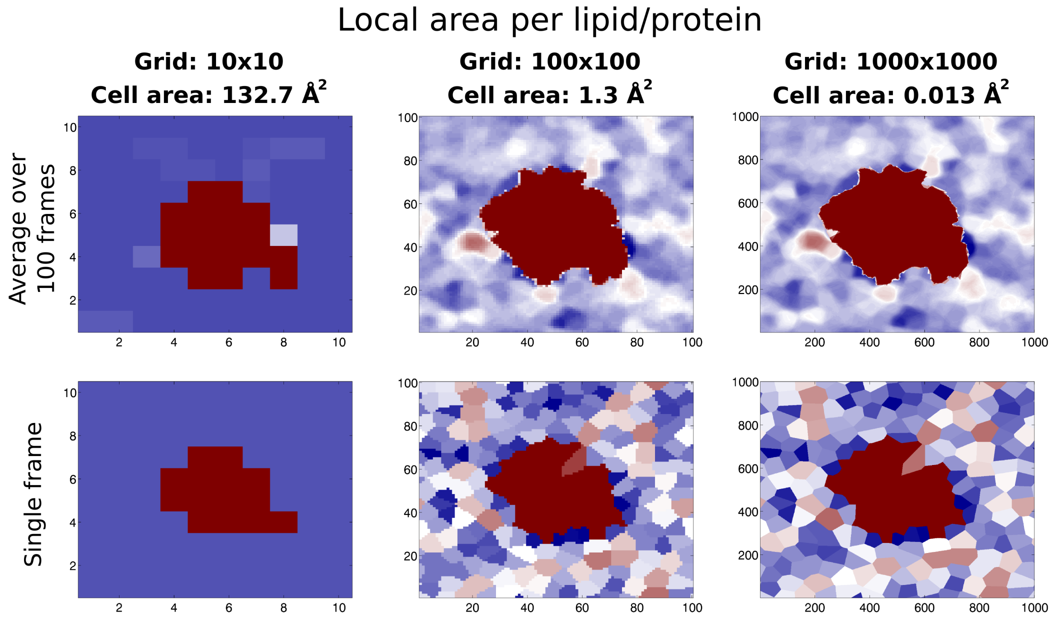

Fig. 4 Effect of increasing the number of grid elements in local area per lipid/protein calculation. Single frame and molecular dynamics trajectory (100 frames) of a VDAC protein embedded in a DMPC bilayer (115×115 Å^2 box area). In case of a single frame analysis, the areas occupied by lipids and protein approach to a Voronoi tesselation. Increasing the number of the grid cells allows reaching a higher resolution of the membrane property mapping. On the other hand, as illustrated for this example in Fig 7 of the main manuscript, areas converge with grid cells smaller than 10 Å^2.

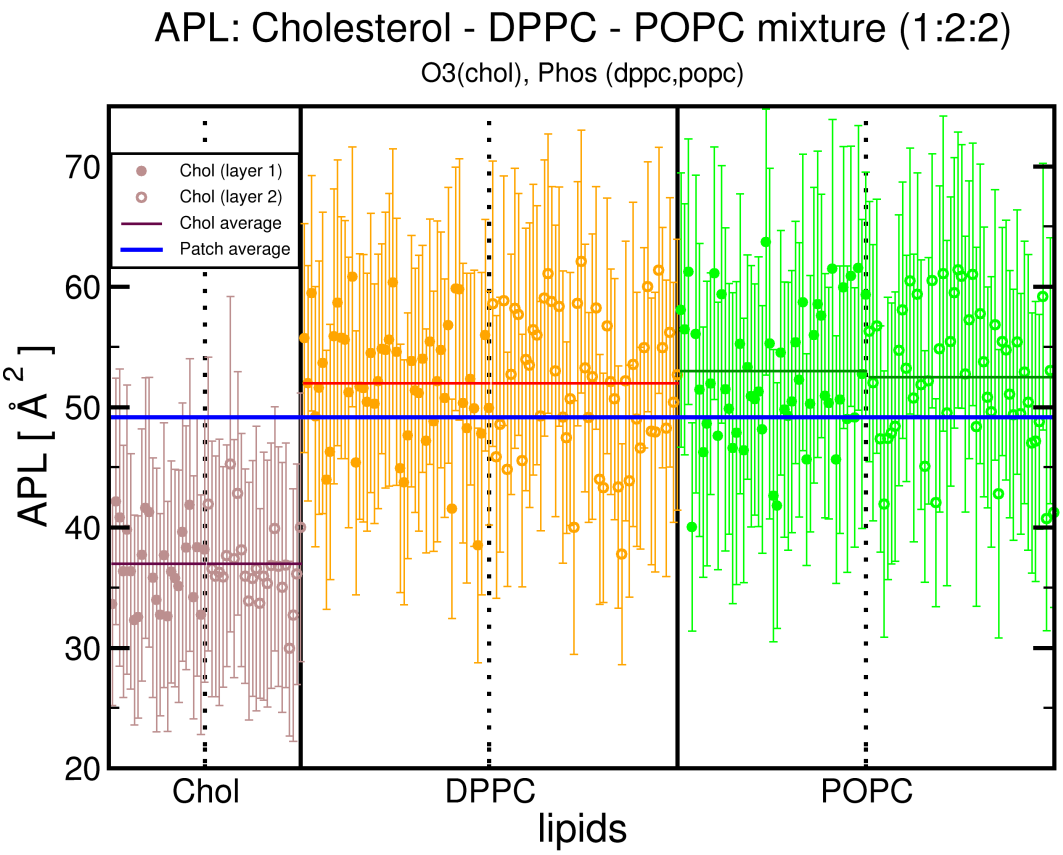

Fig. 5 APL calculated for a Cholesterol:DPPC:POPC mixture (1:2:2). Values correspond to 70 ns of simulation at 310 K, with semiisotropic pressure coupling at 1 bar. Simulation was performed using Charmm36 force field. The lipid patch contained 52 cholesterol molecules. Phosphorus atoms were chosen to represent the phospholipids and alcohol oxygen was selected to represent a cholesterol molecule. The APL averages and standard deviations were calculated for each lipid component and for the whole patch. The average areas for each bilayer component (lealfets 1 and 2) are shown in filled and open circles.

Computational time estimation for the local membrane property analysis

The time requirements for the calculation of different local membrane properties were estimated for the system containing VDAC protein embedded in a DMPC bilayer. The membrane contained 358 lipids. For the calculations 100 frames of the trajectory were used. Performance tests were performed including and excluding the protein group. The protein was represented by 2533 atoms (excluding hydrogens).

The performance of the algorithms was assessed on a workstation with 4 Intel Xeon 2.67GHz CPUs. For the benchmarking of the thickness, APL and curvature calculations, 1 CPU was used. Since the part of the code for the S_{CD} order parameter estimation is parallelized, runs with 1, 2 and 4 CPUs were performed.

Results of the tests are summarized in **Table 1**. Processing of a single frame with the original Gridmat-MD took over 3 minutes. While the calculation of 100 frames of the local thickness, APL and curvature was a matter of tens of seconds, the S_{CD}'s were computationally more demanding. This is explained by the necessity to calculate and map the property for every carbon atom in the acyl chains. However, as illustrated in Table 1, the computational time required for the S_{CD} calculation can be decreased by employing more CPUs, since the problem scales well on a small number of processors.

Table 1 Time requirements for the local membrane property analysis for a 100 frame trajectory containing 358 lipids and a protein represented by 2533 atoms

Analysis	Without protein 1 CPU	With protein 1 CPU	Without protein 2 CPUs	With protein 2 CPUs	Without protein 4 CPUs	With protein 4 CPUs
Thickness	14s	23s	-	-	-	-
APL	17s	26s	-	-	-	-
Curvature	14s	23s	-	-	-	-
S_{CD}	5min 59s	13min 44s	3min 15s	7min 21s	1min 54s	4min 8s

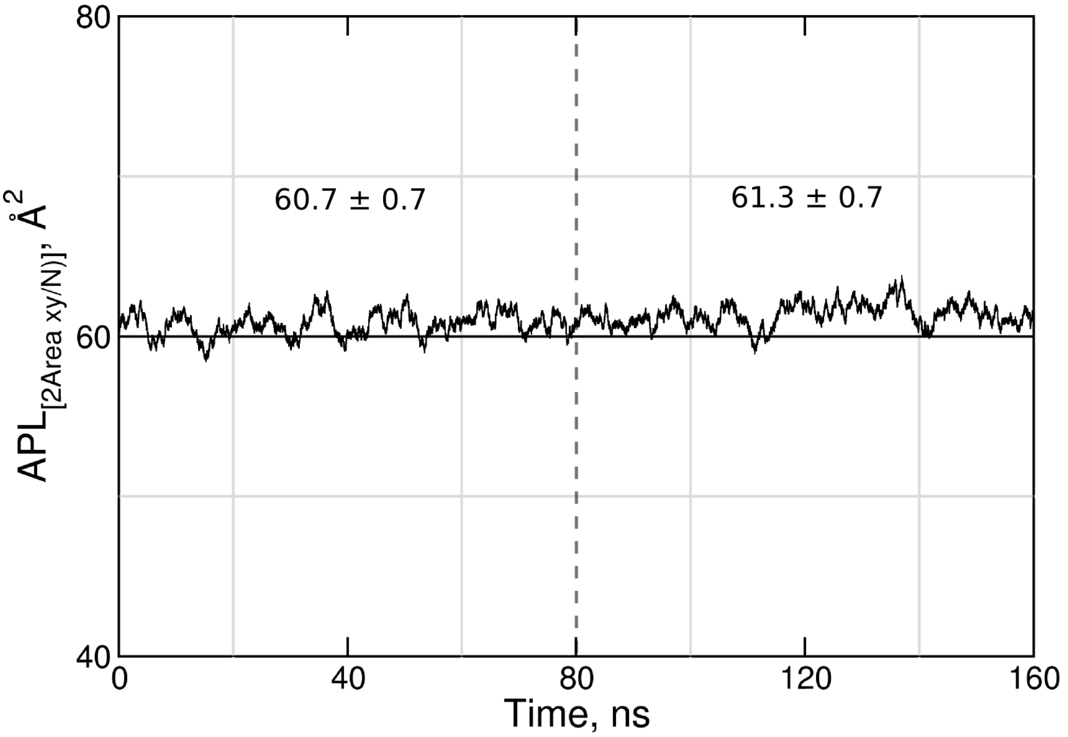

Fig. 6 APL calculated from the simulation box dimensions and lipid number for a pure DMPC simulation. The numbers correspond to the average APL values and standard deviations for each half of the simulation.
